# C1QL1/CTRP14 Is Largely Dispensable for Atherosclerosis Formation in Apolipoprotein-E-Deficient Mice

**DOI:** 10.3390/jcdd9100341

**Published:** 2022-10-06

**Authors:** Hua Guan, Tao Shi, Miaomiao Liu, Xue Wang, Fengwei Guo

**Affiliations:** 1 Department of Cardiovascular Surgery, The First Affiliated Hospital of Xi’an Jiaotong University, Xi’an 710061, China; 2 Shaanxi Key Laboratory of Ischemic Cardiovascular Diseases, Institute of Basic and Translational Medicine, Xi’an Medical University, Xi’an 710021, China

**Keywords:** C1QL1, atherosclerosis, aorta, RNA sequence, ApoE knockout mice

## Abstract

The purpose of this study was to investigate the influence of C1QL1 on atherosclerosis as well as the transcriptomic alteration of the aorta. While complement C1ql-like 1 (C1QL1) is one of the C1q/tumor-necrosis-factor-related protein (CTRP) family members, also known as CTRP14, and is synthesized and secreted mainly by the brain and adipose tissues, the functional properties of the C1QL1/CTRP14 protein outside the brain and adipocytes remain, however, unknown. In this regard, apolipoprotein E (ApoE) knockout (KO) mice were fed a Western diet and injected with adenovirus (Ad) green fluorescent protein or Ad-C1QL1 through the tail vein for 12 weeks. In contrast with the control cohort, the area of atherosclerotic plaque in ApoE KO mice overexpressing C1QL1 showed no significant difference, and the RNA sequence revealed that there were only 111 differentially expressed genes (DEGs) enriched in 26 signaling pathways of the mRNA profile in the aortic atherosclerosis lesions. This analysis also revealed the expression of several genes related to metabolism, organismal system, and human diseases such as type II diabetes, which are not associated with the formation of atherosclerosis in the aorta. These findings illustrate that C1QL1 is largely dispensable for atherosclerosis formation in ApoE-deficient mice and does not improve atherosclerotic plaque formation in the aorta.

## 1. Introduction

The pathological basis of most cardiovascular diseases, such as coronary heart disease, stroke, and myocardial infarction, is commonly induced by the formation of atherosclerotic plaques [1], which involve not only chronic inflammatory reactions but also several effector molecules and complex signaling networks [2,3,4]. Even though patients with cardiovascular diseases (CVDs) exhibit significant improvement after taking statin monotherapy and combining it with other medications, the clinical prognosis is unsatisfactory and poses a major challenge in the clinical treatment process [5]. The identification and discovery of key molecules in the formation of atherosclerosis are, therefore, very important tasks for future development in CVDs and their related therapeutic strategies.

C1q-like 1 (C1QL1), also known as C1q-related factor (CRF) or C1q/tumor-necrosis-factor-related protein 14 (CTRP14), was initially cloned as an emotion-related gene and presents the highest expression level in the brain [6]. C1QL1 has been reported to encode a 258-amino-acid polypeptide with a hydrophobic signal sequence, a collagenous region, and a globular domain at the carboxyl terminus that shares homology with the C1q signature domain [6]. In a previous study, the expression of C1QL1 was essential for the development of hair cell innervation [7,8,9].

Moreover, C1QL1 can specifically bind to the adhesive G-protein-coupled receptor 3 and may participate in synaptic homeostasis, such as synapse formation, maintenance, and elimination [10], so C1QL1 is used to promote migration and proliferation in lung adenocarcinoma [11]. Additionally, Sałkowska et al. revealed that C1QL1 presents a markedly higher level in Th17 cells than in the Th1, Th2, and Treg subtypes, while its protein is well associated with the function of Th17 cells that produce inflammation, which is further linked with atherosclerosis and angiogenesis [12]. Despite this association, the association between C1QL1 and atherosclerosis is unclear.

Based on these findings, this study aims to explore the underlying molecular mechanisms governing C1QL1 regulation of atherosclerosis. ApoE knockout (KO) mice, the most popular animal model for human atherosclerosis, were fed a Western diet and injected with adenovirus (Ad) C1QL1 or Ad green fluorescent protein (GFP) through the tail vein for 12 weeks to confirm this assumption. Lipid profiles and aortic atherosclerosis were evaluated, and RNA sequence analysis of differential gene expression was performed in the aortas. Ad-C1QL1-injected mice and their (Ad-GFP-injected) control counterparts after 12 weeks with the intention of answering two main questions: (1) Does C1QL1 injection through the tail vein affect aortic atherosclerosis and plasma lipids? (2) If not, what is the associated molecular mechanism? The findings of this study demonstrate that, even though the overexpression of C1QL1 elevates high-density lipoprotein cholesterol, C1QL1 is not associated with plaque formation in atherosclerosis.

## 2. Material and Methods

### 2.1. Animals and Diets

In this study, 5 × 10^8^ plaque-forming units of Ad-C1QL1 or Ad-GFP (as a control) were introduced into ApoE KO mice by injection via the tail vein. All mice were nourished by utilizing a Western diet that contained 21% fat and 0.15% cholesterol and were produced by Vital River Company (Vital River Company, Beijing, China). The mice were divided into two cohorts, each comprising fifteen animals, and they were housed in an air-conditioned room for a cycle of 12 h of light and 12 h of darkness, and water and food were allowed *ad libitum*. Eight-week-old male ApoE KO mice were obtained from Vital River Company (Vital River Company, Beijing, China), and pentobarbital sodium (60–70 mg/kg body weight) was injected intraperitoneally to euthanize the mice. The approval of the animal experiment protocol was obtained from the Laboratory Animal Administration Committee of Xi’an Jiaotong University and performed as per both the Guidelines for Animal Experimentation of Xi’an Jiaotong University and the Guide for the Care and Use of Laboratory Animals published by the US National Institutes of Health (NIH Publication number 85-23, revised 2011).

### 2.2. Construction of the Adenoviral C1QL1 Vector and Infection of HEK293 Cells

A recombinant adenoviral vector encoding C1QL1 (Ad-C1QL1) was constructed according to a previously published method [13]. C1QL1 cDNA was subcloned into the adenoviral shuttle plasmid pAdTrack-CMV. Following sequence confirmation, the recombinant shuttle plasmid was transformed into BJ5183 competent cells. The recombinant adenovirus was packaged and amplified in HEK293 cells. Following purification, the viral titer was detected by TCID50. An empty adenoviral vector (Ad-GFP) was constructed as a control.

### 2.3. Biochemical Analyses

Mice were made to fast overnight, after which blood was drawn from the tail vein and was mixed with ethylenediaminetetraacetic acid (EDTA) and then centrifuged at 1500 rpm for 10 min at a temperature of 4 °C to obtain plasma. The plasma sample from each animal was examined in triplicate and measured as per the protocol of the manufacturer utilizing a Benchmark microplate reader (170-6750XTU, Bio-Rad, Veenendaal, The Netherlands). Moreover, high-density lipoprotein cholesterol (HDL-C), low-density lipoprotein cholesterol (LDL-C), and plasma total cholesterol (TC) were analyzed utilizing commercial assay kits (Biosino Bio-Technology & Science Inc., Beijing, China) [14].

### 2.4. Quantification of Atherosclerotic Lesions

To quantify atherosclerosis, pentobarbital sodium (100 mg/kg) was injected intraperitoneally to euthanize the mice, with the aortic trees being opened up and stained using oil red O. The analysis of the size of the en face lesion was performed using an image analysis system (WinRoof Mitani Co., Tokyo, Japan) [14,15]. Moreover, frozen cross-sections were incised at the level of the aortic root to perform a microscopic examination of atherosclerotic lesions. Specifically, the analysis involved ten cross-sections from each mouse, which were then stained using oil red O and hematoxylin–eosin (H&E) to quantify the lesion area. To quantify the area stained with oil red O, an image analysis system (WinRoof Mitani Co., Tokyo, Japan) was employed [16].

### 2.5. Extraction of Total RNA and Construction of a cDNA Library

The extraction of the total RNA from the aortas was performed by using RNAzol (Takara, Tokyo, Japan), whereas Nanodrop (Thermo, Rockford, IL, USA) was utilized to determine the RNA purity and concentration, and the Agilent 2100 Bioanalyzer (Agilent Technologies, Santa Clara, CA, USA) was utilized to verify its integrity. While the purification of mRNA from the total RNA was performed utilizing the NEBNext^®^ Poly(A) mRNA magnetic isolation module (Invitrogen, Carlsbad, CA, USA), the library with an insert size of 400 bp was built utilizing a NEBNext UltraTM RNA Library Prep Kit that adhered to the recommendation of the Illumina manufacturer [17], and the quality of the library was analyzed utilizing the Agilent Bioanalyzer 2100 system. Then, the index-coded samples were clustered on a cBot Cluster Generation System using TruSeq PE Cluster Kit v4-cBot-HS Illumina [18], and library sequencing was executed on an Illumina HiSeq 2500 platform utilizing 100 bp paired-end reads (Illumina, San Diego, CA, USA).

### 2.6. Analysis of Differentially Expressed Genes

The Perl script was employed over 0.25% low-quality bases (Phred quality score < 20) or over 10% Ns for trimming the reads with contaminated adapters. The alignment of clean reads with the mouse reference genome (GRCm38) was subsequently performed using TopHat, whereas the quantification and normalization of gene expression were performed by Cufflinks in RPKM (reads per million per kilobases) [19]. Moreover, DESeq software was used to analyze DEGs and draw a comparison between the Ad-GFP and Ad-C1QL1 cohorts [20], with the false discovery rate (FDR) being employed to set a significant threshold for the *p*-value in various tests. The absolute FDR values of <0.05 and a fold change of ≥2 were used to determine the significance of gene expression, and the DEGs were charted into the Kyoto Encyclopedia of Genes and Genomes (KEGG) datasets to perform the pathway and functional enrichment analysis, with a *p*-value of ≤0.05 being used to determine the significantly enriched KEGG terms.

### 2.7. Real-Time Polymerase Chain Reaction (PCR) Analysis

Total RNA was extracted from the livers of the mice utilizing TRIzol Plus (Invitrogen, Carlsbad, CA, USA), and a SuperScript^®^ III First-Strand Synthesis System (Invitrogen, Carlsbad, CA, USA) was used to synthesize cDNA. The Takara TP800 (Takara Biology Inc., Shiga, Japan) was then used to perform real-time PCR analysis, with the quantification of the number of transcripts and the normalization of each sample being carried out following its β-actin content. The sequences of the PCR primers are illustrated in Supplementary Table S1.

### 2.8. Statistical Analysis

Statistical analyses were executed utilizing either Welch’s *t*-test if the *p*-value was not equivalent or Student’s *t*-test with an equivalent F value. All data are presented as the mean ± standard error of the mean (SEM), and the disparity between the two cohorts was judged to be statistically significant if *p* ≤ 0.05.

## 3. Results

### 3.1. Overexpression of C1QL1 in ApoE KO Mice

**Figure 1:**
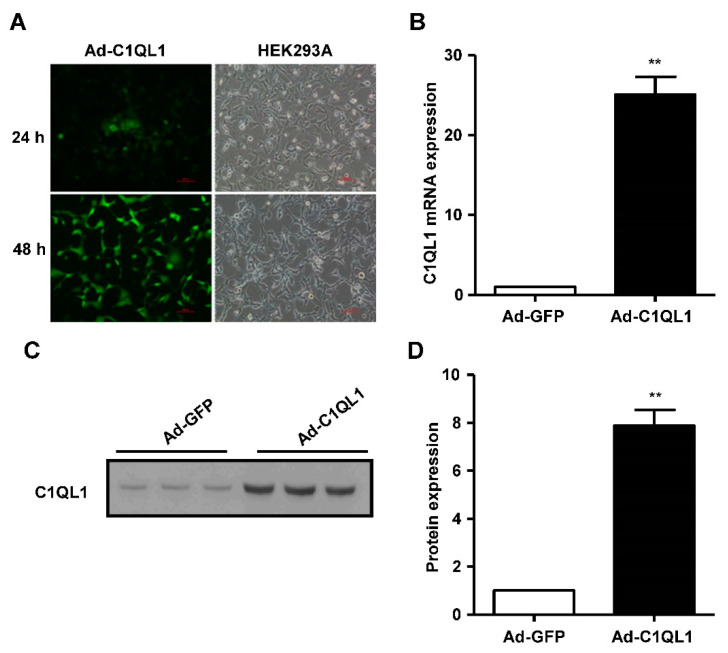
C1QL1 was overexpressed in ApoE KO mice. (**A**) Representative image of adenovirus-infected HEK293A cells. (**B**) RT-qPCR showing the expression of C1QL1 in the Ad-GFP and Ad-C1QL1 cohorts. (**C**) Protein expression of C1QL1 was ascertained by Western blotting (plasma protein was loaded based on the same volume). (**D**) Quantification for C. *n* = 15 of each cohort. Mean ± SEM. ** *p* < 0.01, Ad-C1QL1- versus Ad-GFP-injected mice. Ad-GFP, adenovirus green fluorescent protein; C1QL1, C1ql-like 1; RT-qPCR, real-time quantitative polymerase chain reaction (PCR); KO, knockout.

**Figure 2:**
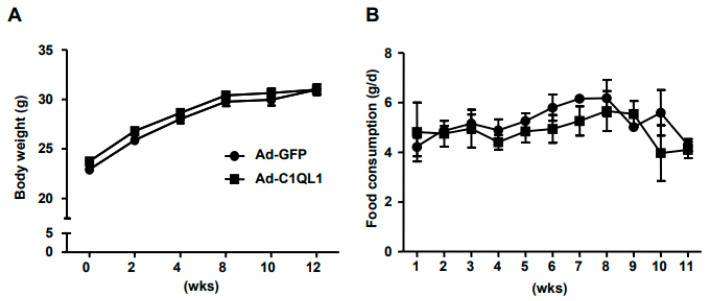
Body weight and food consumption. (**A**) The body weight of ApoE KO mice. (**B**) Food consumption of ApoE KO mice. *n* = 15 of each cohort. Mean ± SEM.

The expression of green fluorescent protein (GFP) was observed 24 h after the transfection of Ad-C1QL1 into HEK293 cells (Figure 1A). While C1QL1 was considerably overexpressed at the protein and mRNA levels in transfected HEK293 cells compared to in the control cohort (Figure 1B–D), eight-week-old ApoE KO mice were treated systemically with adenoviral vectors expressing mouse C1QL1 (Ad-C1QL1) or control Ad-GFP to determine whether administering exogenous mouse C1QL1 affected the formation of atherosclerotic lesions. The plasma C1QL1 protein expression levels were approximately eightfold higher in Ad-C1QL1-injected ApoE KO mice than in Ad-GFP-injected ApoE KO mice six days after systemic administration (Figure 1C,D). Additionally, the outcomes displayed no significant difference in body weight or food consumption between the Ad-GFP- and Ad-C1QL1-injected ApoE KO mice upon their reaching 20 weeks of age (Figure 2A,B).

### 3.2. Overexpression of C1QL1 Elevated HDL-C in ApoE KO Mice

**Figure 3:**
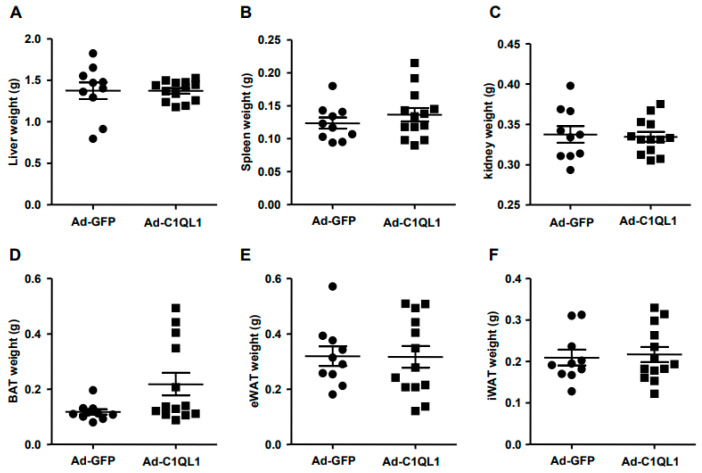
The tissues and organs weight. (**A**) Liver weight; (**B**) Spleen weight; (**C**) Kidney weight; (**D**) BAT weight; (**E**) eWAT weight; (**F**) iWAT weight. Data are presented as the mean ± SEM. *n* = 10 or 13 for each cohort. ApoE KO mice were euthanized, and their tissues and organs were isolated and weighed. BAT, brown adipose tissue; eWAT, epididymis white adipose tissue; iWAT, inguinal white adipose tissue.

We isolated mouse liver, spleen, kidney, brown adipose tissue, visceral adipose tissue, and subcutaneous adipose tissue to analyze the effect of C1QL1 overexpression on the overall tissues and organs of mice. The results showed that, compared with the control group, the overexpression of C1QL1 did not affect these tissues (Figure 3).

**Figure 4:**
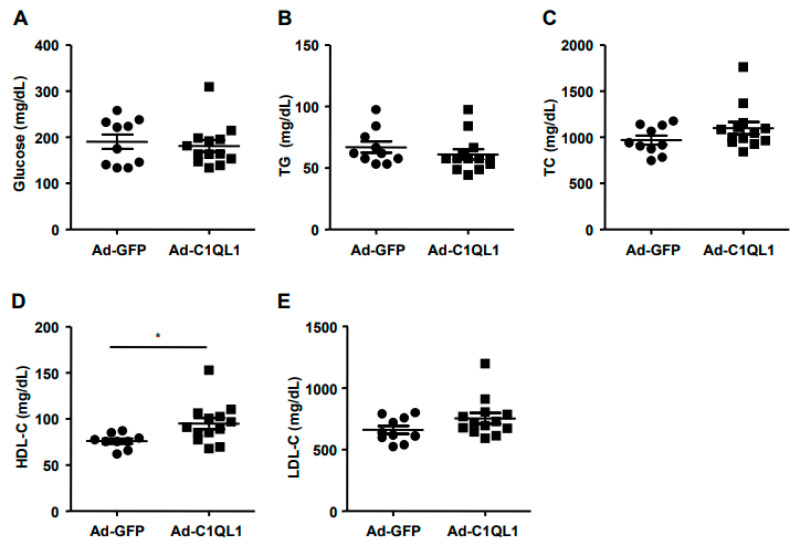
Plasma levels of glucose (**A**), TG (**B**), TC (**C**), HDL-C (**D**) and LDL-C (**E**). Data are presented as the mean ± SEM. *n* = 10 or 15 for each cohort. * *p* < 0.05, Ad-C1QL1 versus Ad-GFP. TG, triglyceride; TC, total cholesterol; HDL-C, high density lipoprotein cholesterol; LDL-C, low density lipoprotein cholesterol.

Additionally, we used a biochemical analyzer to detect the content of TC, TG, HDL-C, LDL-C, and glucose in plasma to analyze the effect of overexpression of C1QL1 on blood indices in mice. As a result, no significant differences were observed in metabolic parameters such as TG, TC, glucose, and LDL-C (Figure 4A–C,E), but we did find that the HDL-C plasma was elevated in the Ad-C1QL1 group as opposed to the Ad-GFP group (Figure 4D).

### 3.3. Increased Production of C1QL1 Did Not Affect the Formation of Atherosclerosis

**Figure 5:**
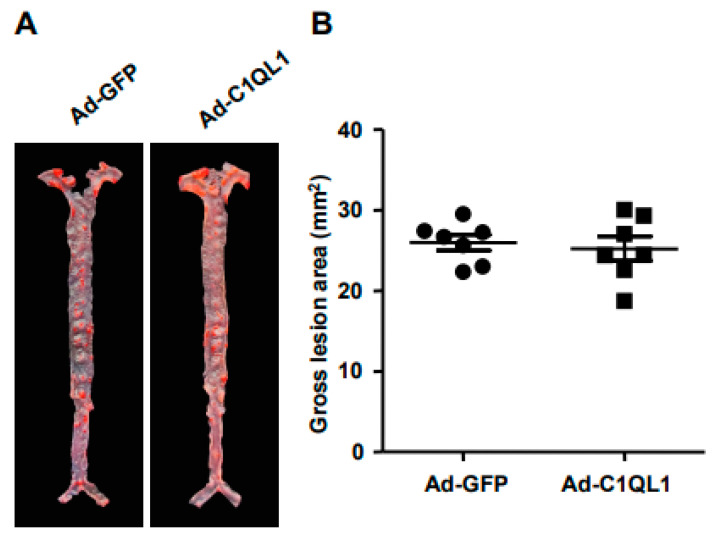
(**A**) Illustrative image of oil red O (ORO) staining in aortas. (**B**)The absolute value of gross lesion area. For each cohort, data are presented as the mean ± SEM. *n* = 10 for each cohort.

**Figure 6:**
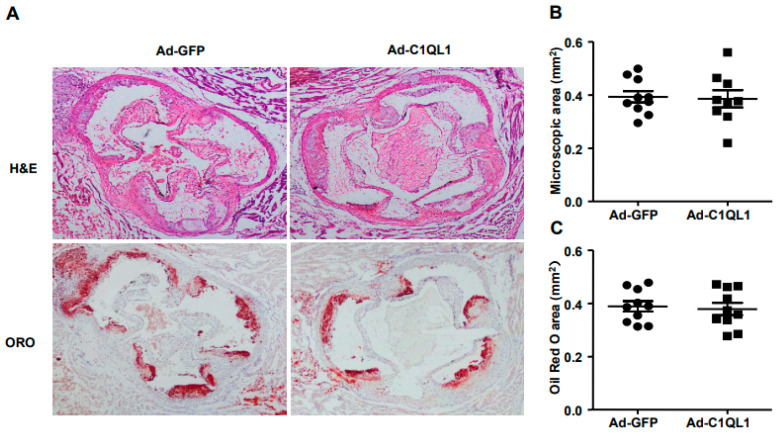
Illustrative micrographs of atherosclerotic lesions of the aortic root. (**A**) Aortic root sections were stained using ORO and hematoxylin–eosin (H&E). (**B**,**C**) Quantitative analysis of aortic root lesion areas is illustrated on the right. *n* = 10 or 15 for each cohort. Data are presented as the mean ± SEM.

In turn, the plaque area was analyzed by oil red O staining after the isolation of the aortic tree to determine the effect of C1QL1 overexpression on the formation of atherosclerotic plaques in ApoE KO mice. The size of the *en face* lesion in the total aorta showed no significant differences between the Ad-C1QL1 and the control cohorts (Figure 5A,B), and the histological examination illustrated that the aortic root atherosclerotic lesions also exhibited no variety in the Ad-C1QL1 cohort. Likewise, microscopic initial lesions in the aortic root were also not significantly different in the Ad-C1QL1 cohort from those in the control cohort (Figure 6B). Consequently, the lipid area in the lesions stained with oil red O also showed no significant differences in the Ad-C1QL1 cohort (Figure 6C).

### 3.4. Gene Expression Profile in the Aorta

**Figure 7:**
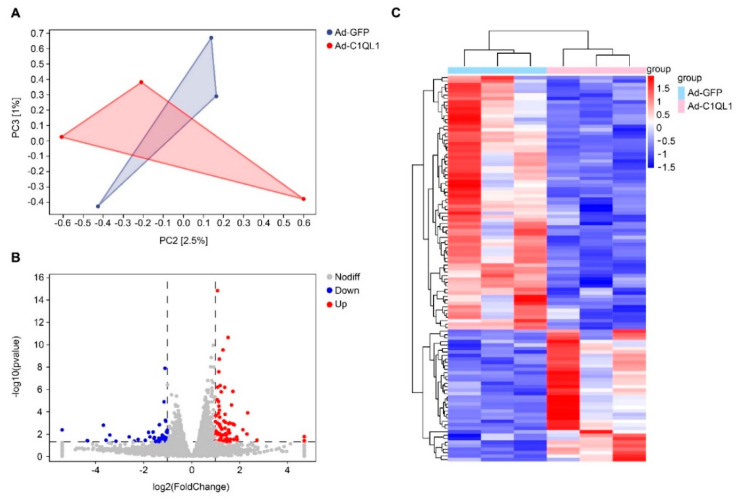
Typical characteristics of DEGs in the aortas of ApoE KO mice. (**A**) Volcano plots show the DEG expression based on RNA sequence analysis. (**B**) Analysis of the DEGs was performed utilizing DESeq software by contrasting the Ad-GFP and Ad-C1QL1 cohorts. (**C**) The expression levels are denoted by colors, red (high expression) and blue (low expression), and are proportional to their brightness (see color bar). *n* = 3 for each cohort.

Considering that the atherosclerotic lesions were not considerably different in the Ad-C1QL1-injected mice, the altered gene expression levels in the lesions were investigated, and RNA sequence analysis was performed on aortic samples from the Ad-C1QL1- and Ad-GFP-injected ApoE KO mice for this comparison. The transcriptomic analysis illustrated that there were 111 DEGs in the Ad-C1QL1 mice as opposed to the control mice, with 73 upregulated genes among these DEGs and 38 downregulated genes (Figure 7B,C and Supplementary Table S2).

As shown in Figure 7A, we performed principal component analysis (PCA) to distinguish differences between aortic samples, and, as a result, the plane figure of PCA between the Ad-C1QL1 and the Ad-GFP groups was partially coincident (Figure 7A), which indicated that the two sets of data were very similar. Cluster analysis was then performed to reveal DEGs in the Ad-C1QL1 group compared to the control group (Figure 7C and Supplementary Table S2). Bearing in mind uncoupling protein 1 (*UCP-1*) is a marker of brown fat nonshivering thermogenesis, *UCP-1* was downregulated significantly after the overexpression of C1QL1 compared to in the control group.

Moreover, cytochrome P450 family 51 subfamily A member 1 (*Cyp51*) is an enzyme that catalyzes many reactions in the synthesis of cholesterol, steroids, and other lipids, and, after injection of C1QL1, *Cyp51* decreased significantly compared to the control group. Moreover, the role of the low-density lipoprotein receptor (*LDLr*) family of proteins has been widely investigated and is related to lipoprotein trafficking [21]. In the present study, *LDLr* mRNA expression was significantly reduced in the Ad-C1QL1 cohort compared to the Ad-GFP group. In turn, Apol is the main apolipoprotein of HDL and participates in the formation of most cholesterol esters in plasma while also promoting the outflow of cholesterol from cells [22], and, in the present experiment, Apol mRNA expression increased significantly after the overexpression of C1QL1. In other words, the migration and adhesion of monocytes, the activation of macrophages, cholesterol metabolism, and the contraction and morphological transformation of smooth muscle cells are closely related to the onset and progression of atherosclerosis in ApoE KO mice [23]. Despite this, in the sequencing results of this study, no related pathway gene expression was observed.

**Figure 8:**
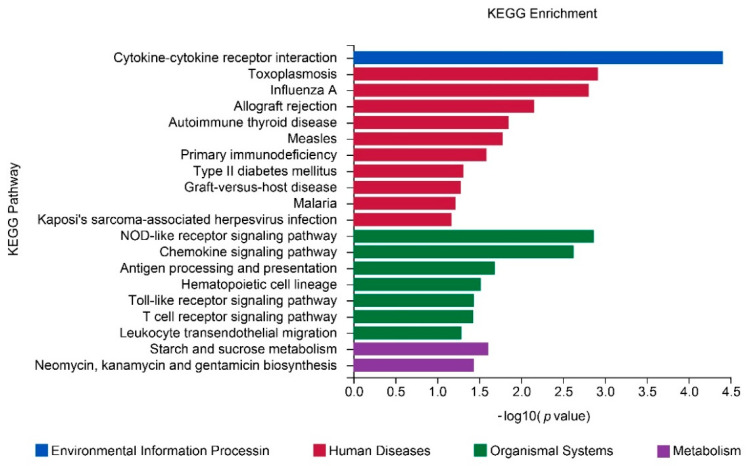
Enrichment KEGG pathways of DEGs in the aorta. The DEGs were charted into the KEGG datasets, and significantly enriched KEGG terms were ascertained by *p* < 0.05. *n* = 3 for each cohort.

The DEGs were grouped into four categories to investigate their functions. The KEGG pathway analysis offered more potentially useful information illustrating the pathways pertinent to the DEGs in the aorta. Based on our DEG outcomes, the KEGG pathway analysis illustrated that these DEGs predominantly belonged to type II diabetes mellitus, the toll-like receptor signaling pathway, the T-cell receptor signaling pathway, and leukocyte transendothelial migration (Figure 8 and Supplementary Table S2).

As illustrated in Figure 8, we analyzed the percentage and the number of DEGs in these signaling pathways, and the outcomes illustrated that the cytokine–cytokine receptor interaction was indeed the most extensive functional pathway, accounting for an aggregated total of seven DEGs (~1% of the total). Additionally, they also illustrated that Ad-C1QL1 does not mainly affect the genes that participate in inflammation and cell migration in the aorta (Figure 8 and Supplementary Table S2).

**Figure 9:**
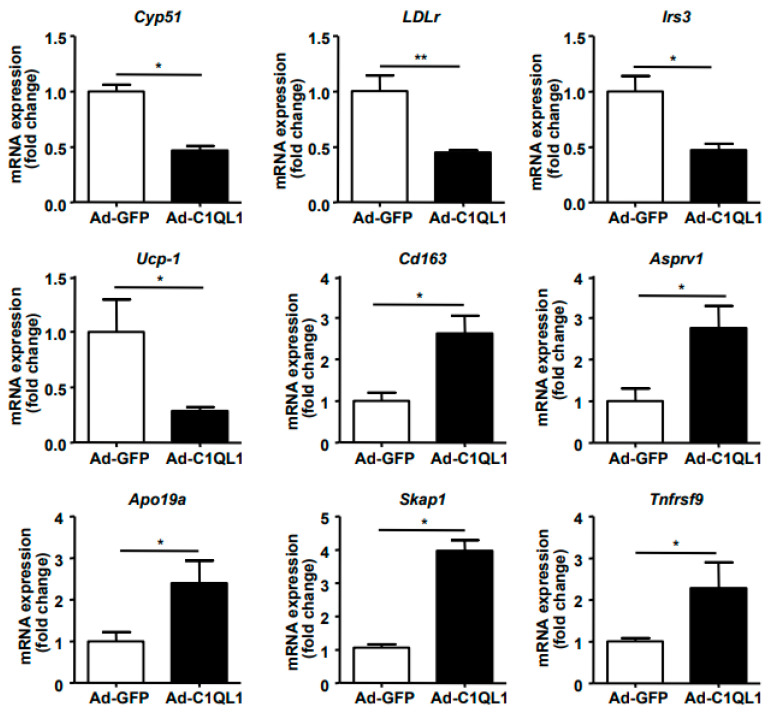
Real-time PCR was performed to identify gene expression in the RNA seq analysis. Genes were randomly selected from all 111 DEGs. *n* = 3 for each cohort. Data are presented as the mean ± SEM. * *p* < 0.05, ** *p* < 0.01, Ad-C1QL1 versus Ad-GFP.

Finally, quantitative (q)RT-PCR analysis of the expression of specific genes in the aorta, including *Cyp51*, *LDLr*, *Irs3*, *UCP-1*, *Cd163*, *Asprv1*, *Apo19a*, *Skap1*, and *Tnfrsf9*, showed results that were consistent with the RNA sequence analysis (Figure 9).

## 4. Discussion

This study demonstrated that overexpression of C1QL1 elevated the plasma HDL-C, but it was not related to aortic atherosclerosis present in ApoE KO mice, nor were significant differences found in plasma TC, TG, LDL-C, and glucose, which, in turn, indicated that the increase in the HDL-C level did not improve the formation of atherosclerotic plaque induced by C1QL1. While there is a possibility that the achieved maximal effects were minimally affected by liver lipid metabolism, the aortic lesions were also not considerably different in the C1QL1 overexpression mice, and this was proven by bioinformatics, specifically the analyzed data of the RNA sequence. Interestingly, a genome-wide association study implicated C1QL1 as a candidate gene associated with total body fat mass [24], indicating that C1QL1 is a potential new therapy for the aetiology of osteoporosis and obesity. However, Sarver et al. demonstrated that C1QL1-deficient mice of either sex gained similar weight and were indistinguishable from wild-type littermates in body composition, lipid profiles, and insulin sensitivity [25]. Hence, C1QL1 is largely dispensable for metabolic homeostasis, but C1QL1 deletion affect physical activity and ingestive behaviours, which was consistent with the present investigation.

Is a high HDL-C level always beneficial? In the present experiment, the plasma HDL-C level was significantly increased compared to that of the control group after Ad-C1QL1 administration, but the area of atherosclerotic plaque of the Ad-C1QL1 group was not reduced (Figure 5 and Figure 6). In epidemiological studies, low plasma levels of HDL-C are associated with an increased risk of cardiovascular disease [26,27,28], and HDL-C has the highest density of all lipoproteins, as well as the highest proportion of protein to lipids [29]. Therefore, this research explored the mechanism by which HDL-C regulates atherosclerosis through a series of experimental studies [30,31,32].

The metabolic pathway of cholesterol reverse transport has attracted extensive attention [33]. By its activation, HDL-C removes cholesterol from lipid cells in the arterial wall and excretes it together with bile in the liver [34], but emerging evidence from studies in animals and humans indicates that high levels of HDL-C are not sufficient to confer atheroprotection and that the functionality of the HDL particles is, in this regard, equally important [35]. Thus, the experimental results of using drugs to upregulate HDL-C in the treatment of atherosclerosis were disappointing.

Therefore, if the HDL subsets with specific protective activity are not changed, the regulation of the HDL level itself may not positively respond to the antiatherosclerotic function [36]. Even though the overexpression of C1QL1 elevated the HDL-C levels in plasma, the plaque formation of atherosclerosis did not decrease due partly to a lack of differences in the practical functionality of HDL.

In this study, RNA sequence analysis of aortas isolated from ApoE KO mice highlighted DEG-enriched signaling pathways, and, even though there was no difference in atherosclerotic plaque formation between the Ad-C1QL1 and Ad-GFP groups, the transcriptome reflected the genes that were actively expressed in the cell and holds the key to understanding the molecular mechanisms of the biological properties. For example, C1QL1 was reported to bind to the brain-specific angiogenesis inhibitor 3, an adhesion type-G protein-coupled receptor (GPCR) that may regulate dendritic morphology by organizing actin filaments [37,38].

We analyzed the DEGs and found that Fam107, which was highly expressed in the C1QL1 overexpression group, plays a key role in actin and microtubule cytoskeleton and negatively regulates focal adhesion assembly, which promotes the migration of malignant glial cells, which was consistent with previous studies and our bioinformatic analogization [11,39]. Moreover, mitogen-activated protein 3 kinases 6 encode a serine/threonine-protein kinase, which forms an integral part of the protein kinase-mediated signal transduction cascade [40]. Additionally, the encoded kinase is involved in regulating the expression of vascular endothelial growth factors [41].

In a previous study, globular C1QL1 directly stimulated the angiogenesis of endothelial cells by activation of the ERK1/2 signaling pathway [42]; specifically, proangiogenic activity was regulated by vascular endothelial growth factor, which is a key regulator in pathological angiogenesis [43]. GPCRs mediate the response of most cells to external stimuli. The ligand binds to the receptor and promotes the exchange of GTP and GDP, resulting in the decomposition of G protein into α and βγ subunits to mediate downstream signals [44]. Typically, C1QL1 is the ligand of GPUR; consequently, there are several encoded DEG transmembrane proteins that act as GPUR, such as Ccr1, Ccr5, P2ry13, Slpr4, and Tent5b.

In conclusion, the overexpression of C1QL1 was not associated with aortic atherosclerosis in ApoE KO mice. Although plasma levels of HDL-C were elevated by Ad-C1QL1 compared to levels in the control group, the plaque formation of the aorta was not ameliorated by increasing HDL-C. Of course, C1QL1 is synthesized and secreted mainly by the brain and adipose tissue. Hence, the functional properties of the C1QL1 protein outside the brain and adipocytes, such as in the aorta, have no effect on inflammation, lipid metabolism, macrophage cholesterol metabolism, or the molecular pathway related to atherosclerosis.

## Abbreviation

AdadenovirusC1QL1C1ql-like 1CRFC1q-related factorFDRfalse discovery rateKOknockoutPCAprincipal components analysisGPCRG protein-coupled bile acid receptor 1CTRPsC1q/tumor-necrosis-factor-related proteinsCVDcardiovascular diseasesCyp51cytochrome P450 family 51 subfamily A member 1GFPgreen fluorescent proteinH&Ehematoxylin–eosinHDL-Chigh-density lipoprotein cholesterolLDLrlow-density lipoprotein receptorLDL-Clow-density lipoprotein cholesterolKEGGKyoto Encyclopedia of Genes and GenomesTCtotal cholesterolTGtriglycerideUCP-1uncoupling protein 1

## Data Availability

The datasets used and/or analyzed during the current study are available from the corresponding author on reasonable request.

## Supplementary Materials

The following supporting information can be downloaded at: https://www.mdpi.com/article/10.3390/jcdd9100341/s1, Table S1: Primers used for real-time RT-PCR; Table S2: The differentially expressed genes of aortas.
