# Supplementary material for: C1QL1/CTRP14 Is Largely Dispensable for Atherosclerosis Formation in Apolipoprotein-E-Deficient Mice
Source: J Cardiovasc Dev Dis. 2022 Oct 6;9(10):341. doi: 10.3390/jcdd9100341 (PMC9604636; doi:10.3390/jcdd9100341)
Supplement: Supplementary file 1 [file jcdd-09-00341-s001.zip › Table S1 Primers used for real-time RT-PCR.pdf]

Table S1. Primers used for real-time RT-PCR

Gene	Accession Number		Sequences（5'-3'）	Products
Apol9a	NM_001162883.1	Reveres	CTTCAGTGCTGCCTCTGACA	245bp
		Forward	GGACCTTGCTGGCAATCCTA	
Asprv1	NM_026414.3	Reveres	CCGGCCTAGCCAGTCTTTAC	107bp
		Forward	ACACTGCTTTAGGCCACCAA	
β-actin	NM_007393.5	Reveres	GCAGGAGTACGATGAGTCCG	74bp
		Forward	ACGCAGCTCAGTAACAGTCC	
Cd163	NM_001170395.1	Reveres	GAGACACACGGAGCCATCAA	161bp
		Forward	CGTTAGTGACAGCAGAGGCA	
C1QL1	NM_011795.3	Reveres	ATTGTCCTTAGCCTGCGTCC	271bp
		Forward	TGGGAAGCTATCCAACCCCT	
Cyp51	NM_020010.2	Reveres	TCTTCACCGAGTCCAAGTGC	209bp
		Forward	GCAGATAGAAGTCGGGAGGC	
Irs3	NM_010571.3	Reveres	TTGGATCTTCGCCCCATTCC	251bp
		Forward	GCCCGAGTTCCAGGAAGAAA	
LDLr	NM_001252658.1	Reveres	CCAATCGACTCACGGGTTCA	109bp
		Forward	ACAGTGTCGACTTCTCTAGGC	
Skap1	NM_001033186.3	Reveres	TACATGTGGCGCTCATCGG	218bp
		Forward	CCATTTCAAAGGCGGTGGTG	
Tnfrsf9	NM_001077508.1	Reveres	CCACAGTGCACCAGATGTGA	263bp
		Forward	TTCTTAAATGCTGGTCCTCCC	
UCP-1	NM_009463.3	Reveres	CACGGGGACCTACAATGCTT	191bp
		Forward	ACAGTAAATGGCAGGGGACG	
