# Supplementary material for: C1QL1/CTRP14 Is Largely Dispensable for Atherosclerosis Formation in Apolipoprotein-E-Deficient Mice
Source: J Cardiovasc Dev Dis. 2022 Oct 6;9(10):341. doi: 10.3390/jcdd9100341 (PMC9604636; doi:10.3390/jcdd9100341)
Supplement: Supplementary file 1 [file jcdd-09-00341-s001.zip › Table S2 The differentially expressed genes of aortas.pdf]

Table S2 The differentially expressed genes of aortas

Name	Genes' description	log2Fold Change	*P*-val
Cyp51	Cytochrome P450 Family 51 Subfamily A Member 1	-1.09	0.000
Acaa1b	Acetyl-CoA Acyltransferase 1	-1.21	0.013
Adamts19	ADAM Metallopeptidase With Thrombospondin Type 1 Motif 19	-2.59	0.019
Adgrb1	Adhesion G Protein-Coupled Receptor B1	-1.43	0.043
Aqp5	Aquaporin 5	-1.55	0.025
Atp5o	ATP Synthase Peripheral Stalk Subunit OSCP	-1.26	0.025
Cilp2	Cartilage Intermediate Layer Protein 2	-1.08	0.029
Clstn3	Calsyntenin 3	-1.35	0.049
Cpn2	Carboxypeptidase N Subunit 2	-1.24	0.026
Dnajb1	DnaJ Heat Shock Protein Family (Hsp40) Member B1	-1.07	0.001
Dsc2	Desmocollin 2	-3.55	0.037
Gabra1	Gamma-Aminobutyric Acid Type A Receptor Subunit Alpha1	-2.21	0.031
Gck	Glucokinase	-3.66	0.00

			2
Gys2	Glycogen Synthase 2	-1.35	0.025
H2bc6	H2B Clustered Histone 6	-1.05	0.022
H3c15	H3 Clustered Histone 15	-1.54	0.021
Hspa1a	Heat Shock Protein Family A (Hsp70) Member 1A	-1.17	0.039
Igsf21	Immunoglobin Superfamily Member 21	-1.92	0.038
Irs3	Insulin Receptor Substrate 3, Pseudogene	-1.06	0.001
Klhl32	Kelch Like Family Member 32	-2.38	0.043
Ldlr	Low Density Lipoprotein Receptor	-1.14	0.000
Map2k6	Mitogen-Activated Protein Kinase Kinase 6	-1.03	0.007
Mgat4c	MGAT4 Family Member C	-4.34	0.040
Myh7b	Myosin Heavy Chain 7B	-3.14	0.045
Myl2	Myosin Light Chain 2	-3.40	0.013
Mylpf	Myosin Light Chain, Phosphorylatable, Fast Skeletal Muscle	-1.48	0.047
Otop1	Otopetrin 1	-1.08	0.043

Pdzrn4	PDZ Domain Containing Ring Finger 4	-1.12	0.019
Popdc3	Popeye Domain Containing 3	-1.59	0.007
Slc8a3	Solute Carrier Family 8 Member A3	-1.01	0.007
Slurp1	Secreted LY6/PLAUR Domain Containing 1	-1.29	0.041
Spon2	Spondin 2	-1.33	0.002
Synpo2l	Synaptopodin 2 Like	-1.00	0.005
Ucp1	Uncoupling Protein 1	-1.78	0.007
Vgll2	Vestigial Like Family Member 2	-4.32	0.041
Wasf1	WASP Family Member 1	-1.07	0.041
Alox15	Arachidonate 15-Lipoxygenase	2.35	0.000
Apol9a	Apolipoprotein L1	1.27	0.013
Arrdc2	Arrestin Domain Containing 2	1.02	0.000
Asprv1	Aspartic Peptidase Retroviral Like 1	1.46	0.012
Bcl11a	BAF Chromatin Remodeling Complex Subunit BCL11A	1.38	0.037
Ccl12	C-C Motif Chemokine Ligand 2	1.73	0.00

			0
Ccl4	C-C Motif Chemokine Ligand 4	1.00	0.043
Ccl6	C-C Motif Chemokine Ligand 15	1.09	0.000
Ccl8	C-C Motif Chemokine Ligand 8	1.13	0.000
Ccr1	C-C Motif Chemokine Receptor 1	1.18	0.000
Ccr5	C-C Motif Chemokine Receptor 5	1.14	0.000
Cd163	CD163 Molecule	1.40	0.000
Cd8a	CD8a Molecule	1.01	0.009
Cd8b1	CD8b Molecule	1.73	0.001
Cfap45	Cilia And Flagella Associated Protein 45	1.47	0.028
Ciart	Circadian Associated Repressor Of Transcription	1.36	0.012
Dbp	D-Box Binding PAR BZIP Transcription Factor	1.67	0.000
Depp1	DEPP1 Autophagy Regulator	1.22	0.003
E2f8	E2F Transcription Factor 8	1.27	0.045
Fam107a	Family With Sequence Similarity 107 Member A	1.17	0.000

Fkbp5	FKBP Prolyl Isomerase 5	1.36	0.001
Galnt15	Polypeptide N-Acetylgalactosaminyltransferase 15	1.08	0.000
Gcsam	Germinal Center Associated Signaling And Motility	1.47	0.046
Gm6133	RPL17-C18orf32 Readthrough	2.73	0.036
Gzma	Granzyme A	1.62	0.030
Gzmb	Granzyme B	1.76	0.031
Gzmk	Granzyme K	1.89	0.019
Hif3a	Hypoxia Inducible Factor 3 Subunit Alpha	1.40	0.003
Ifi211	Myeloid Cell Nuclear Differentiation Antigen	1.09	0.001
Ifi27l2b	Interferon Alpha Inducible Protein 27	1.54	0.005
Ikzf3	IKAROS Family Zinc Finger 3	1.17	0.009
Il10	Interleukin 10	Inf	0.041
Il1rl1	Interleukin 1 Receptor Like 1	1.03	0.006
Il2ra	Interleukin 2 Receptor Subunit Alpha	1.18	0.032
Irf7	Interferon Regulatory Factor 7	1.55	0.00

			1
Lgals3bp	Galectin 3 Binding Protein	1.32	0.000
Lrr1	Leucine Rich Repeat Protein 1	Inf	0.018
Ly6c2	Lymphocyte Antigen 6 Family Member H	1.41	0.001
Lyve1	Lymphatic Vessel Endothelial Hyaluronan Receptor 1	1.02	0.000
Map3k6	Mitogen-Activated Protein Kinase Kinase Kinase 6	1.24	0.000
Mmrn1	Multimerin 1	1.08	0.010
Ms4a4a	Membrane Spanning 4-Domains A4A	1.39	0.000
Ms4a4b	Membrane Spanning 4-Domains A4A	1.78	0.002
Ms4a6b	Membrane Spanning 4-Domains A6A	1.17	0.000
Mt1	Matrix Metallopeptidase 14	1.01	0.001
Nkg7	Natural Killer Cell Granule Protein 7	1.23	0.022
Nr1d1	Nuclear Receptor Subfamily 1 Group D Member 1	1.53	0.000
Oas2	2'-5'-Oligoadenylate Synthetase 2	1.16	0.009
P2ry13	Purinergic Receptor P2Y13	1.18	0.002

Prf1	Perforin 1	2.15	0.004
Rhoh	Ras Homolog Family Member H	1.08	0.009
S1pr4	Sphingosine-1-Phosphate Receptor 4	1.07	0.007
Serpina1e	Serpin Family A Member 1	1.27	0.004
Sh2d2a	SH2 Domain Containing 2A	1.40	0.035
Siglec1	Sialic Acid Binding Ig Like Lectin 1	1.43	0.009
Skap1	Src Kinase Associated Phosphoprotein 1	1.90	0.033
Slc10a6	Solute Carrier Family 10 Member 6	1.20	0.000
Tbx21	T-Box Transcription Factor 21	2.32	0.010
Tent5b	Terminal Nucleotidyltransferase 5B	1.18	0.001
Tnfrsf9	TNF Receptor Superfamily Member 9	1.19	0.037
Usp18	Ubiquitin Specific Peptidase 18	1.06	0.050
Vsig4	V-Set And Immunoglobulin Domain Containing 4	1.02	0.014
Wnt9b	Wnt Family Member 9B	1.60	0.001
